# Are Swedish swingers a risk group for sexually transmitted infections?

**DOI:** 10.1177/0956462420973108

**Published:** 2021-01-09

**Authors:** Nirina Andersson, Jennifer Ejnestrand, Yvonne Lidgren, Annika Allard, Jens Boman, Elisabet Nylander

**Affiliations:** 1 Dermatology and Venereology, Department of Public Health and Clinical Medicine, 174459 Umeå University, Umeå, Sweden; 2 Virology, Clinical Microbiology, 8075 Umeå University, Umeå, Sweden

**Keywords:** Sexual behavior, *Chlamydia trachomatis*, bacterial disease, high-risk behavior

## Abstract

The aim of this study was to investigate whether Swedish swingers constitute a risk group for sexually transmitted infections (STIs). Two swinger clubs were invited to participate. At swinger meetings, members were offered an STI sampling kit and a questionnaire. Samples were analyzed for *Chlamydia trachomatis*, *Neisseria gonorrhoeae*, *Mycoplasma genitalium*, and *Trichomonas vaginalis* using a multiplex real-time polymerase chain reaction assay. In total, 235 swingers participated (118 women and 117 men). Urogenital *C. trachomatis* prevalence was 1.7%. Urogenital *M. genitalium* prevalence was 7.6% for women and 4.3% for men. No one tested positive for *N. gonorrhoeae* or *T. vaginalis*. For women, the mean number of unprotected temporary sex partners within the last 12 months was four men (range 0–35) and three women (range 0–50). Among men, the mean number of unprotected temporary sex partners within the last 12 months was five women (range 0–50) and 0 men (range 0–10). During vaginal sex, 46.6% women and 38.5% men always used protection with a temporary sex partner. Swedish swingers did not seem to have an increased prevalence of STIs. However, there was high-risk sexual behavior with unprotected sex and multiple sex partners, thereby making them a vulnerable group for acquiring STIs.

## Introduction

Infection with *Chlamydia trachomatis* is the most common bacterial sexually transmitted infection (STI) in Europe, with a notification rate of 146 cases per 100,000 population in 2018.^1^ Genital *C. trachomatis* infection may present with burning and discharge, but is often asymptomatic. The infection can cause pelvic inflammatory disease (PID) and damage of the fallopian tubes in women, which in turn can lead to chronic abdominal pain, complications during pregnancy, and infertility.^2^ A correlation between *C. trachomatis* infection and reduced fertility has also been observed among men.^3^ *Mycoplasma genitalium* is also an STI that may cause urethritis and cervicitis, although it is usually asymptomatic. *Mycoplasma genitalium* infection has been associated with PID and infertility in women.^4^ According to a systematic review, *M. genitalium* prevalence in asymptomatic populations ranges from 1.3% in countries with higher levels of development to 3.9% in countries with lower levels.^5^ Infection with *Neisseria gonorrhoe*ae has increased steadily in Sweden since the beginning of 2000, although the incidence is much lower than that for *C. trachomatis*. Apart from cervicitis and urethritis, it can cause PID with a risk of infertility. In men, it can cause epididymitis.^6^ Trichomoniasis is an STI caused by the parasite *Trichomonas vaginalis*. The symptoms are similar to those of other STIs (burning and discharge), but the infection usually occurs asymptomatically. It is the most common non-viral STI worldwide,^7^ with an estimated global prevalence of 5.3% in women and 0.6% in men.^8^ Among young patients visiting an STI clinic in Sweden, the prevalence was 0.16% in women and 0% in men.^9^ Since it is not a reportable disease and surveillance is not generally done, population prevalence in Sweden is unknown.

Transmission of sexually transmitted infections, especially *C. trachomatis* is still not under control in the Swedish population. In 2018, the number of reported cases of *C. trachomatis* was 3127 per 100,000 citizens.^10^ Studies have shown a trend towards riskier sexual behavior with an increased number of sexual partners and more unprotected intercourses.^11,12^ Previous studies have also shown that a small subset of the population (core groups) with large numbers of partners contribute disproportionately to the spread of STIs.^13^ The identification of high-risk groups is essential for STI prevention, and STI programs can be improved by effectively targeting groups at risk.

Swinging can be defined as a non-monogamous behavior in which partners in a committed relationship, as well as singles, engage in sexual activities with others. There are swinger clubs worldwide where people meet to socialize and have sex. These clubs exist in several places in Sweden. In a Belgian study, swinging was associated with sexual risk behavior, and swingers were more likely to be diagnosed with an STI than the general population.^14^ A study including patients at an STI clinic in the Netherlands showed that the STI prevalence was highest in youths, in men who had sex with men, and in swingers.^15^ In the older age group (>45 years), 55% of the STI diagnoses were found among swingers. These swingers had a five times higher prevalence of STI than men and women who were not swingers. Other studies have showed that a minority of swingers use condoms regularly or consistently.^16^ Unprotected sex was more common among drug-using swingers than non-drug-using swingers in a recent Dutch study.^17^ As of today, there is no published study on swingers in Sweden. Enhanced knowledge of Swedish swingers and their sexual risk-taking may identify possible preventive measures in order to reduce the spread of STIs.

We hypothesized that Swedish swingers constitute a risk group in spreading STIs. The purpose of this study was to investigate if Swedish swingers had a higher prevalence of STIs than the general population and patients at the STI clinic, and whether they need information about STIs and safe sex.

## Methods

### Subjects

The two largest Swedish swinger clubs, with approximately 1000 and 2000 members, respectively, were invited to participate in this cross-sectional study which took place from January 2017 to September 2018. At swinger meetings, the club organizer informed the members about the study and handed out packages with written information (about the project and their legal rights as study participants including confidentiality), a questionnaire, an STI sampling kit, including written instructions, and a prestamped return envelope. Participants brought the package with them for testing at home, and were asked to wait one week after the swinger meeting before doing the sampling. The only incentive for participation was being tested for STIs.

### Questionnaire

The questionnaire was in paper form and contained 16 questions that included partner preference (female, male, and both), education level, and gender identity (female, male, or neither). The participants were asked if they were in a permanent sexual relation, the number of sexual partners, and unprotected sexual partners within the last 12 months. Moreover, they reported the type of sexual activity (vaginal, oral, and anal), how often they used barrier protection with a new or temporary sex partner, and if they had acquired an STI within the last 12 months. Participants also reported the duration time as a swinger and the number of swinger meetings they had attended during the last 12 months. Last, they were asked if they needed information and counseling about STIs and safer sex. Participants were also asked to add their telephone number in case we needed to contact them regarding their test results.

### Sampling and laboratory analyses

For women, STI testing was done by using self-collected vaginal and rectal swabs, while men were tested using urine samples. The tests, together with the questionnaires, were sent to the laboratory at the Department of Clinical Microbiology, University Hospital, Umeå, by the participants themselves. The samples were analyzed for *C. trachomatis*, *N. gonorrhoeae*, *M. genitalium*, *T. vaginalis*, *Ureaplasma urealyticum*, *Ureaplasma parvum*, and *Mycoplasma hominis* using a multiplex real-time polymerase chain reaction assay (Anyplex^TM^ II STI-7) according to the instructions from the manufacturer. The reliability of this assay has been confirmed previously.^18,19^ While seven micro-organisms were analyzed, only *C. trachomatis*, *N. gonorrhoeae*, *M. genitalium*, and *T. vaginalis* are reported in this article since the other three are considered colonizing bacteria.

### Positive results

Participants who tested positive for an STI were contacted by telephone. Those with an *M. genitalium* infection were offered a prescription for azithromycin (500 mg day 1 and 250 mg days 2–5). Participants with a *C. trachomatis* infection were directed to their local STI clinic for treatment and contact tracing. Participants testing positive for *N. gonorrhoeae* would also have been directed to their local STI clinic for treatment and contact tracing, while those with a *T. vaginalis* infection would have been offered a prescription for a single dose of 2 g of metronidazole.

### Statistical analyses

The chi-square test was used for comparing prevalence between groups. A *p*-value of 0.05 was considered statistically significant. Statistical analyses were performed using SPSS version 25.0 (SPSS, Armonk, NY, USA: IBM Corp).

### Ethics

The study was approved by the local ethical review board at Umeå University (2014/96-31).

## Results

The study included 235 swingers, of which 118 were women and 117 were men. All 235 participants submitted both questionnaire and STI test. Characteristics of the study population are presented in Table 1. The majority (74.3% of the women and 60.3% of the men) reported 10 or more sexual partners in the last year. Only 8.5% of the women and 18.1% of the men reported four or fewer sexual partners in the last year.

**Table 1.** Characteristics of the study population (*n* = 235).

	Women (*n* = 118)	Men (*n* = 117)
Mean age in years (SD; range)	49 (7; 28–68)	52 (8; 27–69)
Proportion in a permanent relationship	85.1%	80.3%
Mean time as a swinger in years (SD; range)	5.8 (5.1; 0.2–30)	7.1 (6.9; 0–44)
Number of swinger meetings in the last year (SD; range)	9.4 (6.8; 0–40)	9.2 (8.2; 0–55)
Highest level of education completed, %		
Elementary school	3.4	12.9
Upper secondary school	40.2	41.4
University	54.7	43.1
Vocational training programme	1.7	2.6
Proportion having sex with men	100	25.6
Proportion having sex with women	78.0	99.1
Proportion having sex with persons identifying themselves as neither male nor female	0	0.9
Mean number of sexual partners in the last year (SD; range)		
Male	15 (13; 2–60)	1 (2; 0–10)
Female	5 (6; 0–50)	14 (14; 1–100)
Mean number of unprotected sexual partners in the last year (SD; range)		
Male	4 (5; 0–35)	0 (1; 0–10)
Female	3 (6; 0–50)	5 (7; 0–50)

SD: standard deviation.

Ten women (8.5%) and three men (2.6%) reported they had had an STI within the last year. Among women, eight of the STIs were *C. trachomatis* and the other two *M. genitalium*. Among men, two cases were *C. trachomatis* and one man had had an infection with herpes simplex virus.

### Sexually transmitted infection prevalence

The prevalence of urogenital *C. trachomatis* was 1.7% for both women and men. Urogenital *M. genitalium* prevalence was 7.6% among women, compared to 4.3% among men. None of the participants tested positive for *N. gonorrhoeae* or *T. vaginalis*.

Prevalences in the rectal samples (from women only) were *C. trachomatis* 1.7%, *M. genitalium* 6.8%, *N. gonorrhoeae* 0%, and *T. vaginalis* 0%.

Sexually transmitted infection prevalence (*C. trachomatis* and/or *M. genitalium* in urogenital and/or rectal sample) in different subgroups of the swinger population is shown in Table 2. A significantly higher STI prevalence was seen in participants under 45 years of age (*p* = 0.036) and among those not in a permanent relationship (*p* = 0.001).

**Table 2.** STI prevalence (*Chlamydia trachomatis* and/or *Mycoplasma genitalium* in urogenital and/or rectal sample) in subgroups.^a^

			*p*-value
Gender	Women	Men	
	12.7% (15/118)	6.0% (7/117)	0.077
Age (years)	−44	45+	
	16.7% (9/54)	7.2% (13/181)	**0.036**
In a permanent relationship	Yes	No	
	6.3% (12/191)	22.5% (9/40)	**0.001**
Mean time as a swinger (years)	0–4	5+	
	9.0% (9/100)	9.6% (13/135)	0.870
Number of meetings in the last year	0–7	8+	
	11.4% (12/105)	7.7% (10/130)	0.328
University education	Yes	No	
	7.9% (9/114)	10.7% (13/121)	0.454
Sexual partners in the last year	0–9	10+	
	6.6% (5/76)	10.7% (17/159)	0.311
Unprotected sexual partners in the last year	0–3	4+	
	7.3% (8/110)	11.2% (14/125)	0.302
Previous STI within the last year	Yes	No	
	7.7% (1/13)	9.5% (21/222)	0.832
Barrier protection during vaginal sex^b^	Always–usually	Sometimes–seldom–never	
	7.9% (14/177)	14.0% (8/57)	0.168
Barrier protection during anal sex^b^	Always–usually	Sometimes–seldom–never	
	9.9% (11/111)	12.1% (4/33)	0.715
Barrier protection during oral sex^b^	Always–usually	Sometimes–seldom–never	
	0.0% (0/5)	9.7% (22/227)	0.464

STI: sexually transmitted infection. A *p*-value of 0.05 was written in bold.

^a^Total number of study participants = 235, while number of respondents in individual questions may be less because of missing answers.

^b^With new/temporary partner.

Use of barrier protection with a new or temporary partner is shown in Table 3. The participants who always used protection with a new or temporary sex partner comprised less than 50% (regardless type of sexual activity). When comparing those over and under 45 years of age, there was no statistically significant difference in barrier protection always—usually versus sometimes–seldom–never—neither in vaginal nor anal nor oral sex.

**Table 3.** Use of barrier protection with new/temporary partners.

Type of sexual contact	Use of protection—women (*n* = 118), *n* (%)	Use of protection—men (*n* = 117), *n* (%)
Vaginal		
Always	55 (46.6)	45 (38.5)
Usually	36 (30.5)	41 (35.0)
Sometimes	14 (11.9)	14 (12.0)
Seldom	5 (4.2)	8 (6.8)
Never	7 (5.9)	9 (7.7)
Do not have vaginal sex	1 (0.8)	0 (0)
Anal		
Always	40 (33.9)	42 (35.9)
Usually	12 (10.2)	17 (14.5)
Sometimes	6 (5.1)	7 (6.0)
Seldom	2 (1.7)	5 (4.3)
Never	8 (6.8)	5 (4.3)
Do not have anal sex	50 (42.4)	41 (35.0)
Oral		
Always	2 (1.7)	0 (0)
Usually	1 (0.8)	2 (1.7)
Sometimes	2 (1.7)	0 (0)
Seldom	15 (12.7)	9 (7.8)
Never	98 (83.1)	103 (88.8)
Do not have oral sex	0 (0)	2 (1.7)

### Need of information and counseling about sexually transmitted infection

**Figure 1.**
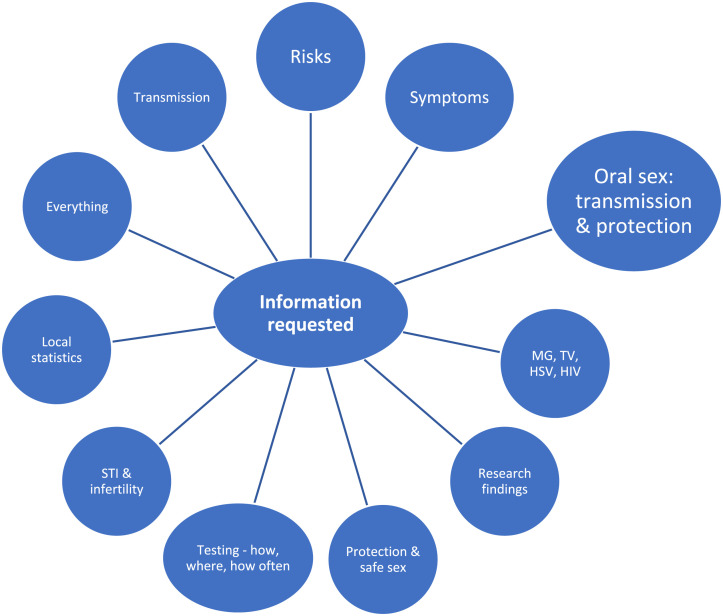
Types of information requested by the study participants.

The participants were asked if they needed information and counseling about STIs. On a scale of 1–6, where 1 = large need and 6 = no need, the mean value among women was 4.1 and 3.8 among men. The participants could comment on what type of information they wished for, and these are shown in Figure 1. Altogether, 40 people wrote a comment, of which 15 (37.5%) asked for information about oral sex (transmission and protection). This kind of information was the most requested.

## Discussion

Our study showed low prevalences of urogenital STIs among Swedish swingers, especially of *C. trachomatis* (1.7% for both women and men), *N. gonorrhoeae* (0%), and *T. vaginalis* (0%). Although STI rates were low, young age and not being in a permanent relationship were risk factors for STIs. Lack of barrier protection during vaginal and oral sex was also associated with higher STI rates, although not statistically significant. We noticed high-risk sexual behaviors with many unprotected sexual contacts and a high number of sexual partners. This can make swinging networks at increased risk for STI transmission should STIs enter the network.

Unlike our urogenital *C. trachomatis* prevalence of 1.7%, a previous study in Sweden that included people who actively ordered home testing kits through a website reported a prevalence of 6.0% among male participants and 4.6% among female participants.^20^ Furthermore, another study of *C. trachomatis* prevalence through home sampling has detected a prevalence of 10.3% among women in Maryland, USA (men were not included).^21^ The studies mentioned above comprise a younger population than in our study, but they have in common that the participants did not actively seek the STI clinic. To compare with a previous study at the University hospital in Umeå, Sweden, the prevalence of *C. trachomatis* infections among people who sought the STI drop-in clinic was 9.9% among women and 11.3% among men.^22^

Urogenital *M. genitalium* prevalence in our study was 7.6% among female swingers and 4.3% among male swingers. In a study by Unemo et al.,^23^ urogenital *M. genitalium* prevalence was 11.0% among women and 9.1% among men who attended an STI clinic in Sweden. While they found a higher prevalence, their group was younger and comprised both symptomatic and asymptomatic patients.

When it comes to *N. gonorrhoeae*, the overall prevalence was 0.27% among females and males in a previous study in the United States.^24^ A prevalence study of people screened for STIs in France showed a prevalence of 1.7% and 3.4% for *T. vaginalis* and *M. genitalium*, respectively. The result did not differ between gender or age-groups.^25^

The vast majority of the participants in our study were in a permanent relationship (85.1% of women and 80.3% of men), and the STI prevalence was significantly lower among them. This might be because those in a permanent relationship limit their sexual networks within the swinging clubs, while the singles may have sexual contacts outside the swinging networks as well. When studying STIs in swingers, the proportion of couples in the study population may influence the results. If one of the persons in a couple acquires an STI when swinging, he or she may infect their partner outside of the swinging environment, and when both partners test positive, the prevalence of swinging-acquired infections in a strict sense may be overestimated among the couples.

The swingers aged >45 years in our study had a lower prevalence of STIs than the younger swingers. These findings differ from a study from the Netherlands where swingers aged >45 years had a higher prevalence of STIs, than both non-swingers aged >45 years and younger swingers.^15^ A previous study from genitourinary medicine clinics in the United Kingdom reported large increases in STIs among older people, and especially noteworthy, both females and males had the highest rates in the 45- to 49-year age-group.^26^ Several studies have also shown less frequent use of condoms with increasing age.^27–29^ This could not be verified in our study, where we saw no statistically significant difference in barrier use between the older and the younger age-groups.

The mean number of sex partners during the last year (women: 15 men and five women, men: 14 women, one man) was higher in our study than that of those in a study about sexual behavior in Sweden from 2007, where the vast majority of women and men had had four or fewer sex partners in the past year.^12^ Only 8.5% of the women and 18.1% of the men in our study had had four or fewer partners. Regarding use of protection with temporary sex partners in the same study, the number of participants who reported always/almost always is similar to those in our study. When comparing the number of participants reporting seldom/never using protecting with a temporary partner, the frequency is lower in our study group. In the abovementioned study, 50.2% of the women and 40.2% of the men seldom/never used protection with a temporary sex partner. Among the swingers in our study, 10.1% of the women and 14.5% of the men seldom/never used protection with a new or temporary partner during vaginal sex. Those always or usually using barrier protection during oral sex comprised only 2.5% of the women and 1.7% of the men in our study. All STIs in our study population was found among those using barrier protection sometimes–seldom–never during oral sex. Due to small subgroups, this was not statistically significant. A recent study from the Netherlands showed that condoms were less used by drug-using swingers than by swingers not using drugs.^17^ Use of drugs was not assessed in the present study.

A trend of higher sexual risk taking in the Swedish population has been observed in a previous study between 1989 and 2007,^30^ which found a significant increase in the prevalence of multiple sexual partners and casual sexual intercourse without the use of protection. Of the sexually active women and men, 7.4% and 11.2%, respectively, reported unprotected casual sex.

Concerning educational level, 54.7% of the women and 43.1% of the men had a university education. According to the Statistical Central Bureau educational attainment report of the Swedish population in 2017, 28% of the population in the age-group 25–34 years (males and females) had completed a university education. The corresponding number was 34% in the age-group 35–44 and 22% in the age-group 45–54 years.^31^ The Swedish swingers in our study tended to have a higher educational level than the Swedish population in general. This might contribute to a higher level of awareness of their sexual health, and thereby resulting in a high frequency of STI testing, early detection, treatment of STIs, and reduced transmission. This could possibly partly explain the findings of low prevalence in our study. In subgroup analysis, swingers with a completed university education had a slightly lower STI prevalence, although this difference was not statistically significant. A negative association between sexual risk behaviors and academic achievement was also seen in the 2009 National Youth Risk Behavior Survey.^32^

Our questionnaire did not assess risk-reducing strategies among the swingers, but one possibility is that the clubs might have rules or recommendations about regular STI testing, thereby achieving low STI prevalences despite high-risk sexual behavior.

We have been able to reach out to active Swedish swingers, with women and men represented evenly. Testing has been performed through home sampling, and people who actively seek the STI clinic have not been included in the study, which may reduce the risk of bias since people who seek the STI clinic more often have symptoms and exposures that put them at higher risk of STIs.

In the present study, we analyzed *C. trachomatis*, *N. gonorrhoeae*, *M. genitalium*, and *T. vaginalis*, but there are no studies on viral STIs in swingers. Existing research on STIs among swingers is limited. Sexually transmitted infection prevalence studies most often constitute a younger population which makes comparison with our study population difficult. The relatively small sample achieved in our study is a limitation for subgroup analyses. Another limitation is the fact that this is a cross-sectional prevalence study with STI testing at only one point in time. However, the answers from the questionnaire have given important information about swinger’s sexual behavior.

In our study, Swedish swingers did not seem to be a population with increased prevalence of STIs. Nevertheless, high-risk sexual behavior occurs in Swedish swinging networks. They have multiple sexual partners, and use of protection is often lacking, thereby increasing their risk of acquiring STIs and related complications. It would be beneficial if sexual health programs and educational material reach out to this subgroup. Furthermore, our results indicate that sexual risk-taking behavior is not confined only to youths but may also be seen in the older population. Frequent STI testing among Swedish swingers could contribute to early detection and treatment, if STIs enter the network.
